# Surface Plasmon Resonance Sensors Using Optical Vortices

**DOI:** 10.3390/nano15120877

**Published:** 2025-06-06

**Authors:** George A. Bulzan, Daniela Dragoman

**Affiliations:** 1 Physics Faculty, University of Bucharest, P.O. Box MG-11, 077125 Bucharest-Magurele, Romania; george.bulzan@imt.ro; 2 National Institute for Research and Development in Microtechnologies (IMT), Str. Erou Iancu Nicolae 126A, 077190 Voluntari, Romania; 3 Academy of Romanian Scientists, Str. Ilfov, Nr. 3, 050044 Bucharest, Romania

**Keywords:** optical vortices, surface plasmon polaritons, sensors

## Abstract

This study investigates the change in both the angular position and width of the reflectance minimum of an SPR sensor in the Kretschmann configuration when optical vortices instead of plane waves are used for illumination. An analytical expression of the reflectance is obtained for incident Laguerre–Gaussian beams, considering only the first-order approximation of the Fresnel reflection coefficient in a Taylor series. Numerical simulations reveal that the detection performance of SPR sensors is practically unaffected if optical vortices of this type are used as sources, even if the topological charges of the vortices are quite large. On the other hand, the use of optical vortices in SPR sensors could be very advantageous for positioning and manipulating analyte molecules on the surface of the sensor.

## 1. Introduction

**Figure 1:**
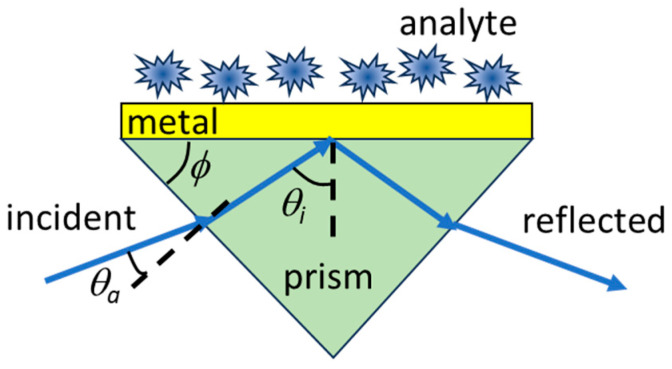
Kretschmann configuration of the SPR sensor considered in this paper.

Surface plasmon polaritons (SPPs) are electromagnetic waves propagating at the interface between media with opposite signs of electric permittivity (typically, dielectrics with positive permittivity and metals, with negative permittivity in the spectral range of interest, in which collective electron modes are excited). SPPs have received increased attention in recent years due to their specific properties, in particular, due to the enhancement of light–matter interactions, as well as due to their wide range of applications in domains such as integrated nano-optics, spectroscopy, imaging, optoelectronics, enhancement of emissive and nonlinear processes, and logic gates [1,2,3,4,5,6,7,8]. Among these applications, one of particular practical interest is in sensing [9,10] a wide range of analytes in the so-called surface plasmon resonance (SPR) configuration consisting of a metal layer deposited on a dielectric substrate, on which the analyte is placed. An SPR in the Kretschmann configuration is illustrated in Figure 1. The SPPs are excited via illumination through a prism and monitored via the reflectance at different incidence angles. More specifically, SPP excitation occurs when the projection of the wavevector of the incident electromagnetic beam along the sensor’s surface equals the SPP wavenumber. The last parameter is extremely sensitive to the refractive index of the dielectric medium/analyte. When the above-mentioned requirement is not met, the metal surface of the SPR strongly reflects the incoming radiation, whereas, if the condition is fulfilled, the reflectance has a minimum. The monitorization of the incident angle for which the minimum reflectance takes place indicates the excitation of SPPs and can thus be used to precisely determine the refractive index of the dielectric medium/analyte. A good SPR sensor should have a minimum reflectance with a low value at a resonant incidence angle and a narrow width around this angle. In our study, the analyte is considered to be placed directly over the metallic layer, although an intermediate dielectric layer between the metal and the analyte could be easily accommodated. Generally, plane electromagnetic waves with transverse magnetic (TM) polarization are used in SPR sensors.

On the other hand, optical vortices (OVs) [11,12,13], as electromagnetic waves with induced phase singularities, have emerged as useful tools in various fields, such as wireless and fiber-based optical communications [14,15,16,17,18], astrophysics [19,20], microscopy [21,22], or particle trapping [20].

The aim of this study is to investigate the working of an SPR sensor when illuminated with OVs instead of plane waves. The reflectance of OVs from a planar surface is expected to have a different expression than that of plane waves. The reason is that, for infinitely-extended plane waves, the reflectance is dependent solely on the incidence angle and the refractive indices of the media on either part of the surface; Snell’s law and Fresnel coefficients adequately describe the reflection of these beams. Instead, the electromagnetic energy flow of finite-width OVs, embodied in the Poynting vector, has a specific helical spatial distribution, and the beam is characterized by a certain angular spectrum distribution. Therefore, although a central incidence angle can be identified, one should specifically take into account the reflection of each angular spectrum component of the incoming beam. The reflectance of OVs can then be defined as a weighted average of each such contribution. Consequently, the reflectance depends not only on the parameters that determine the reflectance of plane waves but also on the angular spectrum distribution of the beam, i.e., on the beam itself.

Previous works on reflection and transmission of OVs from planar surfaces (see, for example Refs. [23,24] and the references therein) have concentrated on the changes in the amplitude and phase profiles of reflected and transmitted beams or on the transverse and longitudinal shifts of the center of gravity of the beam at reflection and transmission. On the contrary, the present study focuses on uncovering the differences between the reflectance of OVs and plane waves from a planar surface and, more specifically, from the surface of an SPR sensor. In particular, we are interested in the changes in the incidence angle at which the reflectance is minimum and eventual modifications of the width of this minimum in order to assess if OVs can be employed as effectively as plane waves in SPR sensors. Such an investigation has not been undertaken up to now, to the best of our knowledge. The problem studied in this work has not only academic merit but is also of practical importance. In particular, this endeavor is justified by the possibility that incident OVs could be used to pin down large molecules (as analytes) on the surface of the SPR sensor, as well as to eventually position them in desired configurations; the trapping and manipulation of nanoparticles by OVs are well-documented facts [25,26,27,28,29,30]. The possibility of stabilizing or immobilizing large molecules on a sensing surface could extend the range of applications of SPR sensors to kinetic processes since such molecules are more prone to undergoing chemical modifications in comparison with free molecules [31]. OVs as light sources for SPR sensors or SPR microscopy could then complement other immobilization techniques [32] in attempts to better understand chemical reactions or biomolecular interactions on a quantitative level [33].

## 2. Methods: Reflectance of Optical Vortices from a Plane Surface

**Figure 2:**
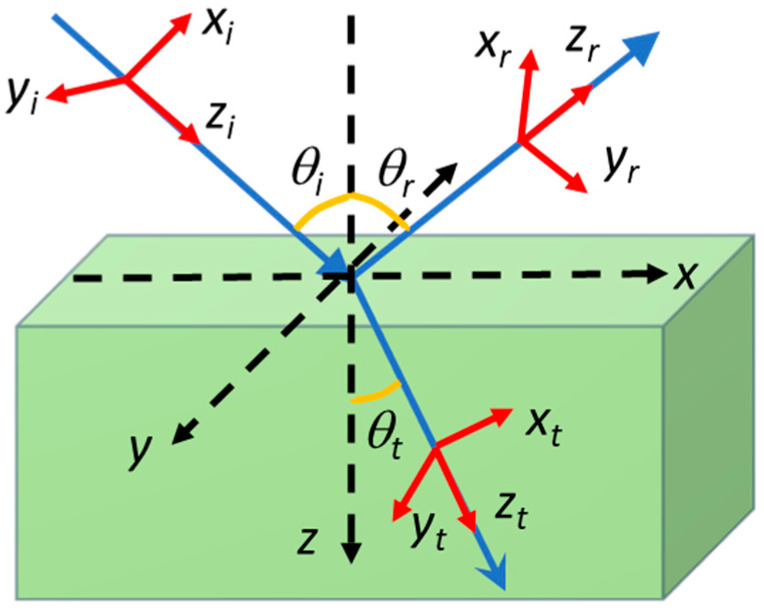
Geometry of OV reflection and refraction at an interface between two media; the global and local coordinate systems are emphasized.

In this study, we specifically refer to Laguerre–Gaussian vortex beams with an integer topological charge *l* that reflect at an interface characterized by Fresnel reflection coefficients *r_TM_* and *r_TE_* for incident beams with transverse magnetic (TM) and transverse electric (TE) polarizations [34]. These reflection coefficients are defined for plane waves; for OVs, they must be applied separately for each plane wave component of the angular spectrum, with specific wavevector components. The geometry of the problem is illustrated in Figure 2, where the subscripts *i*, *r*, and *t* refer to the incident, reflected, and transmitted beams.

Since we are interested in SPR sensing applications, in which the relevant parameter is the reflectance, we focus on the reflected beam only (in any case, the transmitted beam in the configuration in Figure 1 is negligible due to the metal layer). For the types of OVs mentioned above, the reflected and transmitted beams are analyzed in detail in Ref. [23]. More precisely, we consider throughout this study a TM-polarized Laguerre–Gaussian beam, incident on the sensor at the beam waist plane and having a complex amplitude of the form (for *z_i_* = 0):

(1) $$u_{i}\left(l,x_{i},y_{i}\right)={\left[\frac{\sqrt{2}(x_{i}+iy_{i})}{w_{0}}\right]}^{l}exp\left(-\frac{x_{i}^{2}+y_{i}^{2}}{w_{0}^{2}}\right)$$

In Equation (1), $w_{0}$ stands for the beam waist radius. The reflected beam is then calculated by first determining the angular spectrum amplitude of the beam via a two-dimensional Fourier transform. Each angular spectrum component is then individually considered (it is multiplied by the Fresnel reflection coefficient corresponding to the specific wavevector components), and the beam is finally recomposed by performing an inverse two-dimensional Fourier transform. After carrying out a series of lengthy calculations [23], the TM- and TE-polarized components of the reflected field (at *z_r_* = 0) were found to have complex amplitudes given by the following, respectively (see Equations (27)–(31) in [23] for *α* = 1, *β* = 0, *r_TE_* = 0):

(2a) $$u_{r}^{TM}\left(l,x_{r},y_{r}\right)=A_{r}^{TM}\left(l,x_{r},y_{r}\right)u_{r}\left(l,x_{r},y_{r}\right),\cdots \cdots u_{r}^{TE}\left(l,x_{r},y_{r}\right)=A_{r}^{TE}{\left(l,x_{r},y_{r}\right)u}_{r}\left(l,x_{r},y_{r}\right)$$

(2b) $$A_{r}^{TM}\left(l,x_{r},y_{r}\right)=r_{TM}\left[1-\frac{1}{k_{0}}\frac{\partial r_{TM}}{\partial \theta_{i}}\left(-\frac{l\left(ix_{r}-y_{r}\right)}{x_{r}^{2}+y_{r}^{2}}+i\frac{k_{r}x_{r}}{z_{R,r}}\right)\right]$$

(2c) $$A_{r}^{TE}\left(l,x_{r},y_{r}\right)=-r_{TM}\frac{cot\theta_{i}}{k_{0}}\left(-\frac{l\left(iy_{r}+x_{r}\right)}{x_{r}^{2}+y_{r}^{2}}+i\frac{k_{r}y_{r}}{z_{R,r}}\right)$$

(2d) $$u_{r}\left(l,x_{r},y_{r}\right)={\left[\frac{\sqrt{2}(-x_{r}+iy_{r})}{w_{0}}\right]}^{l}exp\left(-\frac{x_{r}^{2}+y_{r}^{2}}{w_{0}^{2}}\right)$$

Here, $k_{0}=k_{i}$ is the wavenumber in the incident medium and $z_{R,r}=k_{r}w_{0}^{2}$, with $k_{r}=k_{i}$ being the wavenumber of the reflected wave. Note that the existence of a TE-polarized component of the reflected field is caused by the distribution of the transverse wavevector component/angular spectrum of the incident field, absent for plane wave illumination. Also, the second term on the right-hand-side of Equation (2b) originates in the first-order approximation of the reflection coefficient in a Taylor series, which is important, especially near the Brewster angle [23,35]; the terms in the Taylor series describe the diffraction corrections of finite-width beams. Based on these expressions, the reflectance can be written as follows:

(3) $$R\left(l\right)=\frac{\int [|u_{r}^{TM}{\left(x_{r},y_{r})\right|}^{2}+{|u}_{r}^{TE}{\left(x_{r},y_{r})\right|}^{2}]dx_{r}dy_{r}}{\int {|u}_{i}{\left(x_{i},y_{i})\right|}^{2}dx_{i}dy_{i}}$$

and can be easily calculated by employing polar coordinates in the transverse planes of both incident and reflected waves. The final result has a simple analytical form, which can be written as follows:

(4) $$R\left(l\right)={\left|r_{TM}\right|}^{2}\left[1+\frac{1}{k_{0}^{2}w_{0}^{2}}\left({\left|\frac{\partial r_{TM}}{\partial \theta_{i}}\right|}^{2}+{cot}^{2}\theta_{i}\right)\left(l+1\right)\right]$$

The expression in Equation (4) can also be used for negative topological charges, with the *l* parameter being replaced by |l| in this case. Note that the formula for R(l) above shows that the reflectance of OVs is always higher than that for incident TM-polarized plane waves, with the difference between the corresponding expressions depending (for a given illumination wavelength) on *l*, $w_{0}$ and the incidence angle.

Moreover, Equation (4), for *l* = 0, also shows that the reflectance of an incident Gaussian beam (see also Ref. [35]) is higher than that of a plane wave, although at the waist plane, as considered in this study, the radius of curvature of the Gaussian beam tends to infinity, i.e., the wavefront is planar. This is yet another example of the different results obtained for plane and Gaussian waves at propagation and/or when interacting with matter. Among examples of such differences, we mention the well-known ABCD law satisfied by Gaussian beams at propagation in the paraxial approximation [36,37], light diffraction from different structures [38], reflection from nonlinear surfaces [39], and the efficiency of nonlinear processes [37,40].

## 3. Results: Optical Vortices as Sources of SPR Sensors

The quite simple and intuitive expression in Equation (4) is the main result of our work. In order to assess the actual difference between the reflectance values of plane waves and OVs in an SPR sensor, we consider in this section a specific example. Namely, we consider a sensor with the same configuration as in Figure 1, in which the metal consists in fact from a succession of Cr and Au layers with thicknesses of 10 nm and 40 nm, respectively, deposited on the prism, and the investigated layer (analyte) has a thickness *t* and refractive indices of *n* of 1.33 or 1.36 (this example can also be relevant for the case when a dielectric layer is placed between the metal and the analyte, with *n* designating in this situation the effective refractive index of the dielectric and analyte layers). We are interested in the eventual change in the position and width of the reflectance minimum, as a function of the incident angle, for different illumination sources; these parameters are used to determine the refractive index of the analyte. In all cases, the wavelength of the incident light is set at 780 nm, and the incidence angle $\theta_{i}$ refers to the angle in the prism. The latter choice renders the simulations more general, since the actual incidence angle from air ($\theta_{a}$) depends on the particular prism angle (*ϕ*), as illustrated in Figure 1. The two angles can be related via the Snell law as follows:

(5) $$n_{a}sin\theta_{a}=n_{p}sin(\phi -\theta_{i})$$

with $n_{a}$ = 1 being the refractive index of air and $n_{p}$ being that of the prism. The Snell law in Equation (5) is obviously fulfilled for plane waves but can also accommodate OVs, as discussed in Ref. [41], for example.

For monochromatic electromagnetic waves, the expression of the reflectivity from both a homogeneous or a stratified medium for an incident TM plane wave ($r_{TM}$) can be found in Ref. [34]. In the case of a stratified medium, as that considered in our example, the Fresnel reflection coefficient $r_{TM}$ is determined in terms of the elements of the 2 × 2 total transfer matrix, also called the Abelès matrix method, relating the components of electric and magnetic fields at different propagation planes/interfaces of the medium.

All simulations in this paper were performed using GNU Octave software, version 5.1.0, which first computes $r_{TM}$ for the stratified structure in Figure 1 using the formalism of Abelès matrices and then implements the analytical formula in Equation (4). The values of the refractive indices at *λ* = 780 nm for all layers were taken from Ref. [42]. The beam waist radius was chosen as $w_{0}=10\lambda$, with larger values of this parameter rendering the differences between plane waves and OVs less significant.

**Figure 3:**
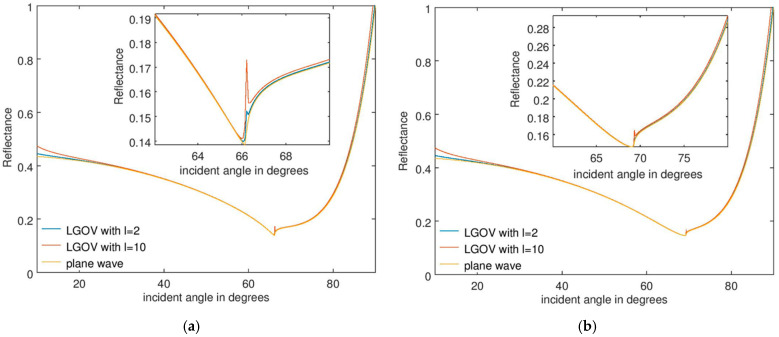
Reflectance dependence on the incident angle for an SPR sensor consisting of a fused silica prism and an analyte with a thickness of 60 nm and a refractive index of (**a**) 1.33 and (**b**) 1.36.

**Figure 4:**
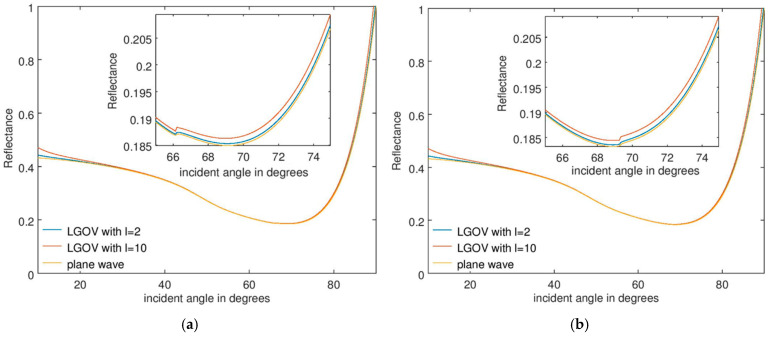
Reflectance dependence on the incident angle for an SPR sensor consisting of a fused silica prism and an analyte with a thickness of 150 nm and a refractive index of (**a**) 1.33 and (**b**) 1.36.

The numerical results are presented in Figure 3a and Figure 3b for a fused silica prism, when the refractive indices of the analyte are *n* = 1.33 and 1.36, respectively, for a thickness *t* = 60 nm. Figure 4 illustrates the corresponding results for *t* = 150 nm. The most important observation is that the angular position and width of the minimum reflectance are practically the same when the sensor is illuminated with plane waves or OVs, with these parameters being crucial in sensing applications. For *t* = 60 nm, a small and very narrow increase in *R* is observed near the reflectance minimum, no longer discernible at higher *t* values. This discontinuity is caused by the fact that only the first-order approximation of the reflection coefficient was considered in Equation (2). As mentioned above, discontinuities are expected to appear near the Brewster angle and to diminish if higher-order approximations of the Fresnel reflection coefficient are taken into account.

A full Taylor expansion of reflected Laguerre–Gaussian beams, valid for both paraxial and nonparaxial regimes, and specifically applied to calculate the transverse and longitudinal shifts of the center of gravity of the beams at reflection, is in fact given in Ref. [43]. As expected, the simulations in this reference show that there are also discontinuities/sharp changes in both types of shifts near the Brewster angle and that, as the number of terms in the Taylor expansion increases, the numerical results become closer to the precise/non-approximated ones. Although in our case only the first-order approximation of the reflection coefficient was used, the largest value of the extremely abrupt discontinuity, illustrated in Figure 3a, is of only 3%. This small value justifies our choice of not including in the analysis higher-order terms in the Taylor expression of the Fresnel reflection coefficient. Indeed, as inferred from the full Taylor expansion of this parameter in Ref. [43], the use of higher-order terms would render the analytical calculation of the reflectance much more difficult. The insightfulness of the relatively simple expression in Equation (4) would be lost, without gaining a significant increase in simulation precision.

It should be emphasized that discontinuities near the Brewster angle of various parameters of a TM-polarized Laguerre–Gaussian beam cannot be discounted since at this angle, splitting, rotation, and spiraling of polarization components of such beams have been theoretically predicted and observed (see, for example, Ref. [24]). They are caused by the coupling of the complex spatial beam profile with the longitudinal Goos–Hänchen shift due to diffraction effects and the transverse optical spin–Hall shift caused by spin–orbit interaction. The first is produced by the gradients of plane wave components of the Laguerre–Gaussian beam situated in the plane of incidence, while the latter originates in the different geometric phases acquired by the slightly rotated planes of incidence of the out-of-plane angular components. The modifications in the amplitude and phase profiles of Laguerre–Gaussian beams due to coupling with these transverse and longitudinal shifts depend on the topological charge *l* and the beam waist.

The results in Figure 3 and Figure 4 also show that the angular position of the discontinuities in the reflectance remains practically the same when the thickness *t* of the analyte layer increases, although the reflectance minimum shifts towards larger incidence angles as a result of a change in the sensed material.

**Figure 5:**
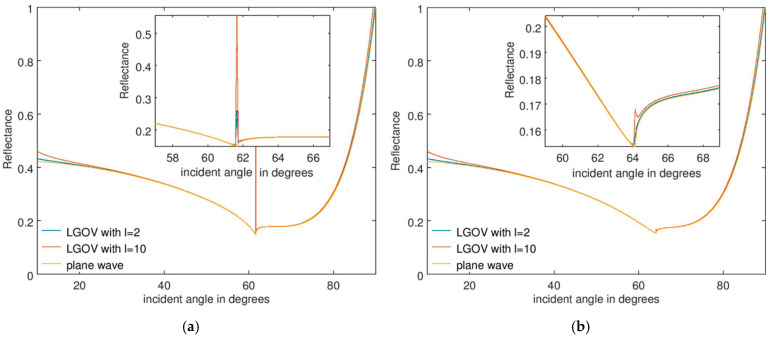
Reflectance dependence on the incident angle for an SPR sensor consisting of a BK7 prism and an analyte with a thickness of 60 nm and a refractive index of (**a**) 1.33 and (**b**) 1.36.

**Figure 6:**
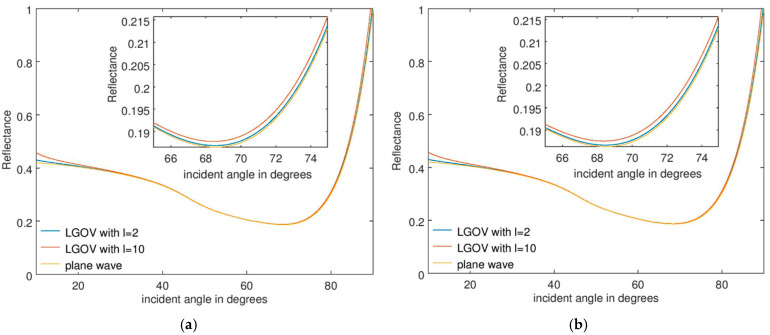
Reflectance dependence on the incident angle for an SPR sensor consisting of a BK7 prism and an analyte with a thickness of 150 nm and a refractive index of (**a**) 1.33 and (**b**) 1.36.

The same general behavior is found for other prism materials, for instance, for a BK7 glass (see the corresponding Figure 5 and Figure 6), with the difference from the previous simulations being that, in this case, the shift in the reflectance minimum is more pronounced. In particular, for *t* = 150 nm, the discontinuity in *R* is no longer located near the reflectance minimum.

In all simulations, as indicated by Equation (4), the difference between the reflectance for plane waves and OVs increases with *l*, irrespective of the material/refractive index of the prism. Also, for both fused silica and BK7 prisms, the positions of the discontinuities in *R* do not change with *t* for illumination with OVs but depend slightly on the refractive index of the analyte. This dependence indicates extremely shallow penetration of the incident, evanescent electromagnetic wave in the analyte layer.

## 4. Conclusions

The study reported in this work responds to the question of the nature of change in the reflectance minimum of an SPR sensor in a Kretschmann configuration when illuminated with OVs instead of plane waves. Considering only the first-order approximation in the Taylor series of the Fresnel reflection coefficient for TM-polarized waves, an analytical expression for the reflectance of OVs has been obtained. This expression clearly identifies the influence of different parameters, such as topological charge and waist radius, on *R*. In all cases, the reflectance of OVs was found to be higher than that for plane waves, with the difference between them increasing as *l* increases and $w_{0}$ decreases. However, simulation results of a specific example show that the angular position of *R* remains practically the same, although the minimum reflectance value increases slightly with the topological charge *l*. From a practical point of view, this increase is hardly noticeable, even for *l* values as large as 10. This result indicates that the detection performances of an SPR sensor are not downgraded by illuminating it with OVs instead of plane waves. On the other hand, the use of the former types of electromagnetic waves is highly desirable when the analyte has to be precisely positioned and/or manipulated on the surface of the SPR sensor. The dimension of the molecule that must be manipulated determines the appropriate topological charge value of the incident OVs. The waist radius of these beams also influences the ability to accurately immobilize analyte molecules on the surface of the SPR sensor, especially for lower values of this parameter.

## Data Availability

The authors confirm that the data supporting the findings of this study were generated using the method, software, and parameters indicated within the article.
